# Synergism With ε-Polylysine Hydrochloride and Cinnamon Essential Oil Against Dual-Species Biofilms of *Listeria monocytogenes* and *Pseudomonas lundensis*

**DOI:** 10.3389/fmicb.2022.885502

**Published:** 2022-06-10

**Authors:** Junli Zhu, Jingcong Liu, Xiaoli Hong, Yang Sun

**Affiliations:** College of Food Science and Biotechnology, Zhejiang Gongshang University, Hangzhou, China

**Keywords:** *Listeria monocytogenes*, *Pseudomonas lundensis*, ε-polylysine hydrochloride, cinnamon essential oil, dual-species biofilms

## Abstract

Various pathogenic and spoilage bacteria frequently coexist in meat processing environments and can form multispecies biofilms, causing significant health and economic issues. Despite the prevalence and coexistence, only less is known about possible interactions between *Listeria monocytogenes* (LM) and spoilers like *Pseudomonas* species, and their community-wide resistance against natural preservatives. This study evaluates the interactions between mono- or dual-species biofilms formed by LM and *Pseudomonas lundensis* (PL), as well as the sensitivity of these bacteria in dual-species biofilms to ε-polylysine hydrochloride (ε-PLH) alone or combined with cinnamon essential oil (CEO). The results showed that the biofilm cell density of *P. lundensis* in dual species was higher (*p* < 0.05) than LM, constituting about 85% of the total population. More biofilms and exopolysaccharide both in mono- or dual species of the two psychrotrophic strains were greatly produced at 15°C than at 30°C. The biomass, biovolume, and thickness of dual-species biofilms were significantly lower than single PL biofilm when tested using crystal violet staining, confocal laser scanning microscopy, and scanning electron microscopy, indicating the competitive interactions between them prevail. Additionally, ε-PLH significantly reduced the biofilm development as mono- and dual species in a concentration-dependent manner, especially single LM biofilm, which was consistent with the decrease in autoinducer-2 (AI-2) activity. LM as dual-species biofilms exhibited lower sensitivity to ε-PLH than its mono-biofilm probably due to protective effect conferred by PL. ε-PLH in combination with CEO, at the maximum sublethal concentrations (MSCs), showed enhanced inhibitory activity against dual-species biofilm formation, as evidenced by thin spare spatial structures and reduced AI-2 activity. In addition, the preformed dual biofilms were dramatically eradicated following treatment with ε-PLH combined with CEO at higher than minimum inhibitory concentration in comparison with either of the compounds used alone, indicating the synergistic antibiofilm of the two preservatives. This study reveals the competitive interactions between the two strains in dual-species biofilms, in which the dominant PL significantly contributed toward the tolerance of LM to ε-PLH, and the use of combined preservatives shows it is an effective strategy to control the multispecies biofilms in meat processing.

## Highlights

- LM and PL exhibited a competitive interaction in dual-species biofilms.
- Dominant PL in dual-species biofilms enhanced the resistance of LM to ε-PLH.
- ε-PLH combining with cinnamon EO had synergistic antibiofilm of the dual-species.
- Inhibition of two natural preservatives was correlated with repression of AI-2.
- Combination ε-PLH with cinnamon EO could be a promising approach against multi-biofilms.

## Introduction

A biofilm consists of microbial cells embedded in a matrix of self-produced extracellular polymeric substances (EPS) on biotic or abiotic surfaces. A serious problem in the food industry is the formation of microbial biofilms that can harbor and transmit pathogenic and spoilage bacteria. Approximately more than 60% of foodborne outbreaks are linked to the presence of biofilms in food processing environments (Piercey et al., 2016). *Listeria monocytogenes* (LM) is a psychrotrophic, Gram-positive, and facultative anaerobic bacterial pathogen that is responsible for listeriosis in humans. Presence of LM on food products may be attributed to its survival and proliferation at low pH, high salinity, and chilling temperatures. Additionally, the ability of LM to form biofilms on food contact surfaces has been demonstrated in previous studies (Gandhi and Chikindas, 2007; Rodriguez-Lopez et al., 2018; Liu et al., 2020). Sessile cells are less susceptible to disinfectants than planktonic cells, which makes it hard to eliminate them and allows them to manage to survive in food processing or storage environments for prolonged periods (Giaouris et al., 2013; Pang et al., 2019). However, the capability of LM to form mono-species biofilm does not seem to be a key factor determining their persistence in food processing (Fagerlund et al., 2021).

The resident microbiota in food processing plants can affect the growth of LM. Psychrotrophic *Pseudomonas* spp. are the dominant bacteria that cause spoilage in aerobically chilled meat (Stanborough et al., 2018; Wickramasinghe et al., 2019). *Pseudomonas lundensis* (PL), which is considered one of the most prevalent meat-spoilage bacteria, produces fluorescent and pyocyanin pigments, off-odors, and slime (Wickramasinghe et al., 2019; Fang et al., 2022). Moreover, PL has high capacity to produce biofilms, and a great amount of biofilm was formed at low temperatures (Liu et al., 2015). In a cold chain, the residential psychrotrophs, such as LM and *Pseudomonas*, attach to the surfaces of fresh meat products and spread through growth, ultimately resulting in the increase of cross-contamination, which leads to shortened shelf-life, and foodborne illnesses (Tenderis et al., 2021).

In food processing environments, biofilms are generally composed of dynamic microbiota, which we refer to as multispecies biofilms. During the formation of multispecies biofilms, the complex interactions within the community significantly affect the structure and biological function of biofilms (Giaouris et al., 2013). More recent biofilm studies have examined LM in co-culture with other food-related commensal bacteria or foodborne pathogens in mixed-species biofilms. Norwood and Gilmour (2000) demonstrated higher LM numbers in mono-cultures compared with the multispecies biofilms formed after its association with *Staphylococcus xylosus* and *Pseudomonas fragi*. *Pseudomonas fluorescens* may enhance the tolerance of LM to antimicrobial in dual-species biofilms by bacterial suspension co-culture (Puga et al., 2016). Thus, once the spoilage and pathogenic bacteria in the food environment form complex multispecies biofilms, they showed enhanced survival and tolerance to chemical disinfectants and UV radiation due to the production of EPS and microbial interactions in co-culture (Bridier et al., 2015; Wang et al., 2020).

Recently, effective natural antimicrobial compounds have received interest as an alternative strategy to inhibit the biofilm formation compared with some chemical sanitizers. ε-Poly-lysine hydrochloride (ε-PLH) as a homopolymer of ε-poly-lysine has been found to exhibit good antimicrobial activity with low price and higher stability than ε-poly-lysine (Ding et al., 2019; Li et al., 2021). At present, the effective antimicrobial properties of ε-poly-lysine against wide microorganisms are well documented (Hyldgaard et al., 2014); however, there are few studies on the inhibitory activity of ε-PLH and ε-poly-lysine on the planktonic and biofilm cells of foodborne pathogens. In addition, cinnamon essential oil (CEO) has a wide variety of secondary metabolites that exhibits antibacterial properties. Its main active components include cinnamaldehyde, cinnamate, and cinnamic acid, demonstrating a high performance to control biofilm formation of various pathogenic bacteria and spoilage-related bacteria (Vasconcelos et al., 2018; Liu et al., 2020). The combination of essential oils with other antimicrobial agents to inhibit biofilm-forming bacteria is now a hot research topic, to enhance efficacy, decrease adverse sensory effects, and reduce the required dose, such as binary EOs (Vázquez-Sánchez et al., 2018), combining with nisin (Abdollahzadeh et al., 2014), and ε-PLH (Li et al., 2021).

The objective of this study was to investigate the dual-species biofilms of foodborne pathogen LM and spoilage bacterium PL, to explore the interactions between the two strains under co-cultivation conditions, and their sensitivity to ε-PLH. Furthermore, the synergistic effects of ε-PLH in combination with CEO on the formation and eradication of dual-species biofilms were further evaluated. The results not only shed new light on the significance of properties and interactions in multispecies biofilms from meat products, but also uncovered the impacts of combining natural antimicrobial compounds on multispecies biofilms.

## Materials and Methods

### Bacterial Strains and Media

*Listeria monocytogenes* ATCC19112 used in this study was purchased from the China Microbial Preservation Center (Beijing, China). The strain of PL 28 was previously isolated from chilled beef in our laboratory (Fang et al., 2022). These strains stored at −80°C were revitalized on tryptic soy broth (TSB, Bio-Tech, Qingdao, China) at 30°C with consecutive transfers after 24 h shaking incubation (180 rpm).

### Counting of Planktonic and Biofilm Cell Populations

Briefly, overnight activated cultures of the two strains were diluted at a ratio of 1:1,000, with 5.0 log cfu/ml as the final cell concentration for each species. The diluted LM and PL were inoculated separately or mixed in tube or polystyrene plates with TSB, and the inoculated samples were cultivated statically for 24 and 48 h at 30 and 15°C. For enumeration of planktonic cells, bacterial suspension as mono- or dual species from each tube was serially diluted with sterile peptone water (0.85% NaCl and 0.1% peptone), and then aliquots of 0.1 ml were spread on Pseudomonas CFC Selective Agar (Bio-Tech, Qingdao, China) for PL and PALCAM Agar (Bio-Tech, Qingdao, China) for LM. For enumeration of the biofilm cells, mono- and dual-species biofilm cells were cultivated on the surface of stainless steel coupons (SS, AISI304, 1 cm × 1 cm × 0.2 cm) in six-well polystyrene plates (one coupon in each well) (Wang et al., 2020). The coupons were soaked in 70% ethanol for 5 min and autoclaved at 121°C for 15 min prior to the experiment. Wells containing TSB alone were considered as control. After incubation at 30 and 15°C, SS coupons were taken out and washed three times with phosphate buffered saline (PBS, pH 7.0) to release loosely attached cells. Subsequently, the biofilm cells on the SS surfaces were obtained by vigorously vortexing in 0.1% peptone water for 5 min. The homogenized suspension was then diluted and enumerated on the above two selective agar plates, and the total number of colonies was counted after incubation at 30°C for 24 h.

### Crystal Violet Biofilm and Extracellular Polysaccharides Assay

The activated LM and PL as mono- or dual-species biofilms were quantified using crystal violet (CV) assay. Briefly, the above diluted cultures were inoculated in 96-well polystyrene microtiter plates as mono- or co-culture, and uninoculated TSB was used as negative control. Following incubation, the biofilm in each well of microtiter plates was carefully washed thrice with sterile PBS to remove unattached cells. Biofilm cells on the bottom and side of each well were stained with 0.2% (w/v) CV for 15 min. After a second washing step, biofilm cell-associated CV in each well was resolubilized with 95.0% ethanol (v/v) for 5 min. The optical absorbance of the samples was measured at 590 nm using a microplate reader (Infinite 200, Tecan, Switzerland).

Extracellular polysaccharides in biofilm were extracted according to Wang et al. (2020). The above diluted LM and PL containing about 5 log cfu/ml of the two strains as mono- or dual species were transferred into six-well polystyrene microtiter plate and incubated at 30 and 15°C for 24 or 48 h. After the cultures in each well were carefully aspirated, the biofilm cells were washed with sterile PBS, redissolved with PBS, and sonicated at 50 kHz for 5 min. Bacterial pellets were then removed by centrifugation (8,000 × *g* for 30 min) and filtering by a 0.22 μm membrane, and the extracting supernatant was added with 5% phenol and sulfuric acid and bathed at 100°C for 10 min. The absorbance value was determined at 490 nm to quantitatively measure exopolysaccharides using glucose as the standard.

### Confocal Laser Scanning Microscopy and Scanning Electron Microscopy Observation

The biofilms formed by PL and LM as mono- or dual species were further observed by optical microscopy (OM), confocal laser scanning microscopy (CLSM), and scanning electron microscopy (SEM). After incubation for 24 h at 30°C, the glass slips (8 mm × 8 mm) in six-well polystyrene microtiter plates were taken out and rinsed with PBS three times to remove the planktonic bacteria. The biofilm cells on the surface were then stained with 0.2% (w/v) CV and directly observed using an OM (Olympus CX21, Tokyo, Japan). Meanwhile, the biofilm structures of mono- or dual species were examined using CLSM with objective lens (Carl Zeiss LSM710, Jena, Germany) after stained with 0.3% SYTO-9 and 0.3% PI (Sigma-Aldrich, Shanghai, China) in the dark at 30°C for 15 min. The maximum excitation/emission used for these stains was approximately 505/525 for SYTO-9 (green channel) and 493/635 for PI (red channel). Representative CLSM images from each coupon were acquired by scanning z-stacks at a scanning step size of 1 μm and were processed in IMARIS 7.6 software (Bitplane AG, Zurich, Switzerland). Three representative areas were selected by scanning the total surface area with a 20× objective. The Zeiss confocal software was used to analyze the biofilm images, allowing for collection of z-stacks. Quantitative structural parameters of the biofilms, including bacterial biovolume and thickness, were calculated using PHLIP (Almeida et al., 2003).

In addition, the biofilms formed by PL and LM as mono- or dual species were further observed by SEM. After the removal of culture media and washing, the glass slips incubated for 24 h at 30°C were collected and fixed with ice-cold 2.5% glutaraldehyde overnight at 4°C. After washing, biofilm cells on glass slip were post-fixed with 1% osmium tetroxide for 1 h. The samples were washed in PBS (pH 7.0) and dehydrated with a graded series of ethanol solutions from 30 to 100% for about 15 min at each step. The dehydrated samples were coated with gold-palladium and observed using SEM (Hitachi SU8010, Japan).

### ε-Polylysine Hydrochloride Treatment

ε-Polylysine hydrochloride (>90% pure, w/w, Silver-Elephant Bio-engineering Co., Ltd., Zhejiang, China) was prepared as a stock solution (20 mg/ml) in distilled sterile water and diluted to work solution (5 mg/ml). First, minimal inhibitory concentration (MIC) and maximum sublethal concentrations (MSC) values of ε-PLH against PL and LM in TSB were obtained using the microwell dilution assay (Wang et al., 2020). MIC was defined as the lowest concentration of the antimicrobial that inhibited >90% of the growth of the tested microorganism (Oo et al., 2010). MSC was considered as the last tested concentration that allowed bacterial growth compared with control (Di Pasqua et al., 2006). Then, the diluted cultures of LM and PL were inoculated separately or mixed in TSB, which were supplemented with ε-PLH at the different concentrations of 0, 2, 4, 8, 16, 32, 64, and 128 μg/ml at the initial time. After incubation at 30°C for 24 h, the populations of biofilm cells as mono- or dual species were counted as mentioned in the “Counting of Planktonic and Biofilm Cell Populations” section, and biofilm biomass and extracellular polysaccharides were determined using the above CV assay and phenol-sulfuric acid method as described in the “Crystal Violet Biofilm and Extracellular Polysaccharides Assay” section, respectively.

### ε-Polylysine Hydrochloride Combined With Cinnamon Essential Oil Treatments

The inhibitory activity of ε-PLH in combination with CEO on the formation of dual biofilms of LM and PL was further analyzed. CEO was obtained from Borui Spice Oil Co., Ltd. (>95% pure, v/v, Jiangxi, China). The diluted LM and PL cultures were mixed in equal volumes for dual-species inoculation in fresh TSB, with 5.0 log cfu/ml as the final cell concentration for each species. The two preservatives at the MSCs using alone or combination, including ε-PLH at 8 (P8) or 16 μg/ml (P16), CEO at 25 (C25) or 50 μg/ml (C50), and four combined groups (P8 + C25, P8 + C50, P16 + C25, P16 + C50), were supplemented to wells of the microtiter plate. After incubation at 30°C for 24 h, biofilm biomass and extracellular polysaccharides of the multispecies biofilms were determined. Then, CLSM and SEM were utilized to observe the effect of ε-PLH combined with CEO on the spatial structure of the dual biofilms.

In addition, ε-PLH and CEO were further tested against 24 h performed dual biofilms formed by LM and PL in microtiter plates for their ability to reduce the CV biomass (biofilm removal, %) and metabolic activity (biofilm inactivation, %). Briefly, the 24 h preformed biofilms in each well of 24-microtiter plates were carefully removed and washed with PBS (pH 7.0) to remove unattached cells. ε-PLH and CEO either alone or combination at high than MICs were supplemented to the preformed biofilm in the microtiter plates, including ε-PLH at 64 (P64) or 128 μg/ml (P128), CEO at 50 (C50) and 100 μg/ml (C100), four combined groups (P64 + C50, P64 + C100, P128 + C50, P128 + C100), and sterile peptone water as control. After treatment at 30°C for 30 min, the biomass of inhibitors exposed and non-exposed biofilms was quantified by CV staining. The percentage of biomass removal (% BR) was determined by Equation 1, where is the OD value for preservative non-exposed biofilms (control, C) and T is the OD value for inhibitor exposed biofilms.


(1) %BR=(C-T)/C×100

The metabolic activity of preservative exposed and non-exposed biofilms was evaluated using XTT [2,3-bis (2-methoxy-4-nitro-5-sulfophenyl)-2H-tetrazolium-5-carbox-anilide] assay kit (Jiangsu Kaiji Biotechnology Co., Ltd., Nanjing, China). After incubation at 37°C for 4 h in the dark, the absorbance in each well was measured at 450 nm. The percentage of biofilm inactivation (% BI) was determined according to Equation 2, where C is the absorbance for control and T is the absorbance for inhibitor exposed biofilms.


(2) %BI=(C-T)/C×100

Additionally, the effects of ε-PLH combined with CEO on the spatial structure of the 24 h performed dual biofilms were further examined by CLSM.

### Autoinducer-2 Detection

According to Bodor et al. (2008), autoinducer-2 (AI-2) activities of PL and LM as mono- or dual species were assayed using *Vibrio harveyi* BB170. Overnight culture of *V. harveyi* BB170 was diluted (1:5,000) in a fresh AB medium, and the diluted cells were dispensed into 96-well microplates, fresh AB medium as blank control. After incubation for 24 h at 30°C, the supernatant of mono and co-culture treated with ε-PLH and CEO at the MSCs was centrifuged (10,000 × *g*, 3 min), and then filtered through a 0.22 μm membrane filter. Each cell-free filter was added to each well of microplates containing the diluting BB170 strain. After incubated at 30°C with shaking, the bioluminescence of *V. harveyi* BB170 was monitored every 30 min using a luminometer (PerkinElmer Victor X, PerkinElmer Inc., Waltham, MA, United States), continuous for 6 h.

### Statistical Analysis

All the experiments were performed in three biological replicates, and the mean and standard deviation of experimental values were calculated. The figures were processed using Prism 8.0 software (GraphPad Software Inc., La Jolla, CA, United States), and statistical significance was assessed by one-way ANOVA method using SPSS 17.0 software package (SPSS Inc., Chicago, IL, United States). Significant differences among experimental groups of mono- and dual cultures treated with or without ε-PLH and CEO are expressed as *p* values of <0.05* and <0.01^**^.

## Results

### Mono- and Dual-Species Planktonic and Biofilm Cells

The planktonic and biofilm cell population of LM and PL as mono- and dual-species cultures incubated at 30°C or 15°C are described in Table 1. The planktonic cell number of single LM reached 9.0 log cfu/ml during the stationary phase, which was 0.2 log cfu/ml higher than in the co-culture (*p* > 0.05). The planktonic population of PL in both mono- and dual-species cultures reached about 9.0 log cfu/ml in TSB. In addition, LM and PL strains were able to form mono-specie biofilm on the surface of SS, but their biofilm cell count of each strain was significantly lower than the corresponding planktonic cell (*p* < 0.05). In dual-species biofilms, PL attained approximately 0.6 log cfu/cm^2^ higher cell density compared to LM, thus constituting approximately 85% of the total population both at 30 and 15°C. Moreover, there was significant difference of LM or PL biofilm cell count between mono- and dual-species biofilms (*p* < 0.05). Biofilm cell densities of LM in dual-species biofilms reached 6.47–6.58 log cfu/cm^2^ at 30 and 15°C, in contrast to about 7.0 log cfu/cm^2^ in mono-species biofilm (*p* < 0.05). The dual-biofilm cell populations of PL were 7.26–7.40 log cfu/cm^2^ during mature phase, which were slightly lower than mono-species-attained numbers of biofilm cells (*p* < 0.05).

**TABLE 1:** Planktonic cell and biofilm cell of *L. monocytogenes* and *P. lundensis* in mono-culture and co-culture at 30 and 15°C on stainless steel coupons.

		Planktonic cell (log cfu/mL)				Biofilm cell (log cfu/cm^2^)			
		Mono-culture		Co-culture		Mono-culture		Co-culture	
		LM	PL	LM	PL	LM	PL	LM	PL
30°C	24 h	9.07 ± 0.16	9.05 ± 0.14	8.83 ± 0.17	9.01 ± 0.12	7.01 ± 0.07	7.71 ± 0.11	6.58 ± 0.18*	7.40 ± 0.08*
	48 h	8.98 ± 0.03	9.04 ± 0.06	8.71 ± 0.20	8.87 ± 0.14	6.75 ± 0.14	7.43 ± 0.19	6.46 ± 0.15*	7.17 ± 0.20
15°C	24 h	8.02 ± 0.25	8.21 ± 0.18	7.87 ± 0.09	8.12 ± 0.13	6.25 ± 0.12	7.00 ± 0.12	5.95 ± 0.10*	6.71 ± 0.13
	48 h	9.04 ± 0.06	9.10 ± 0.28	8.82 ± 0.07	9.04 ± 0.19	6.94 ± 0.06	7.61 ± 0.13	6.47 ± 0.13*	7.26 ± 0.16*

*All values are expressed as log cfu/ml or cm^2^ ± standard deviation (n = 5).*

Significantly different with regard to mono- or co-culture, *p < 0.05.

**FIGURE 1:**
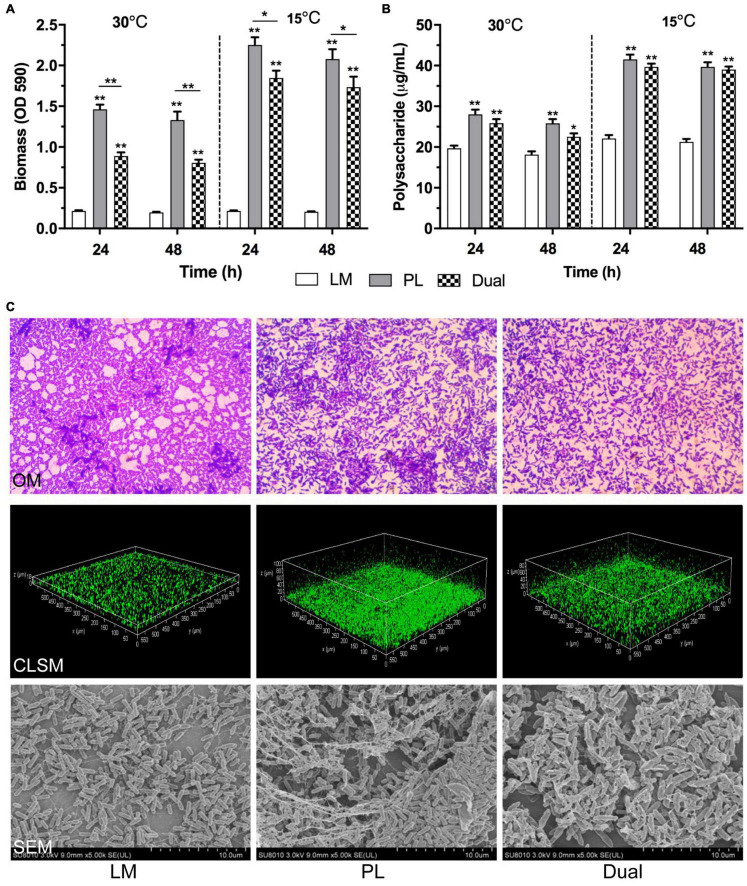
Biofilm formation of *L. monocytogenes and P. lundensis* as mono- and dual species at 30 and 15°C. **(A)** Biofilm biomass by CV, **(B)** polysaccharides production, and **(C)** OM images (1,000×), CLSM staining living cells by SYTO9 (20×) and SEM images (4,000×). Data were expressed as mean ± standard deviations (*n* = 3). *p < 0.05; **p < 0.01: significant interspecies difference.

Moreover, PL showed significantly higher quantities of biofilm biomass (Figure 1A) and exopolysaccharides (Figure 1B) than LM in mono species (*p* < 0.01) using microtiter plates, indicating the strong biofilm-forming ability of PL. In spite of no difference of viable cell number, biofilm biomass and exopolysaccharide both in mono- or dual-species LM and PL were greatly higher at 15°C than those at 30°C, indicating that the low temperature could stimulate the secretion of biofilm extracellular matrix of the two strains. Unexpectedly, the levels of biofilm formation and polysaccharide production in the co-culture were significantly lower than that of mono-species PL both at two culture temperatures (*p* < 0.05).

Meanwhile, OM, CLSM, and SEM were employed to visualize the adhesion and structure of mono- and dual-species biofilms at micro level (Figure 1C). Massive adhesive cells were distributed in the mono-species biofilm of LM and PL after 24 h by OM observation. Compared to LM, PL formed the denser and thicker maturing biofilm with multiple layers followed by CLSM and SEM analysis, while the dual-species biofilms developed sparer and higher heterogeneity than PL. In addition, LM and PL as mono-culture, and their co-culture formed the biofilms with thickness of 20.3, 100.5, and 90.4 μm, respectively. Furthermore, PL as mono- or dual species produced the large EPS wrapping cell aggregation forming spatial structure observed by SEM, especially mono-species PL. Generally, the results of these microscopies were consistent with those as determined by CV staining and exopolysaccharides.

### Effect of ε-Polylysine Hydrochloride on Mono- and Dual-Species Biofilm Cells

**FIGURE 2:**
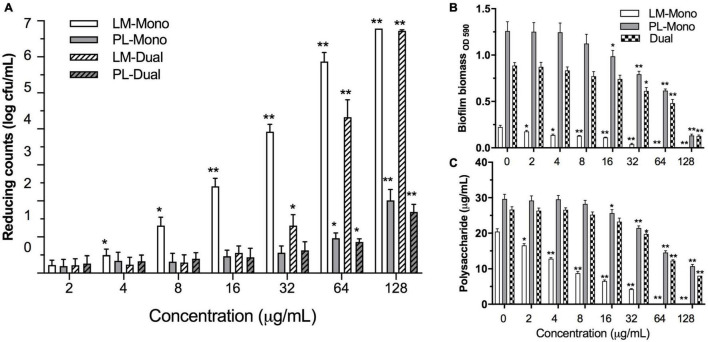
Effects of ε-PLH on the biofilm formation of *L. monocytogenes and P. lundensis* as mono- and dual species at 30°C for 24 h. **(A)** Reducing biofilm cells, **(B)** biofilm biomass by CV, and **(C)** polysaccharides production. Data were expressed as mean ± standard deviations (*n* = 3). *p < 0.05; **p < 0.01: significant interspecies difference.

The MICs of ε-PLH against LM and PL were 16 and 100 μg/ml in TSB, respectively (Supplementary Table 1). ε-PLH at the concentration lower than 8 and 50 μg/ml had no significant effect on the planktonic cell densities of LM and PL during the stationary phase, respectively, and these latter concentrations of 8 and 50 μg/ml were thus as MSCs of ε-PLH against the two strains. Effects of ε-PLH treatment on the biofilm formation of the two strains, cultivated under either mono- or dual-species conditions on SS coupons, were evaluated (Figure 2A). The treatment of ε-PLH resulted in a significant reduction of mono- or dual-species biofilm biomass of LM and PL compared with untreated control (*p* < 0.05). The biofilm cell counts of dual-species LM were apparently decreased after 32 μg/ml ε-PLH treatment compared with mono-species LM treated with 4 μg/ml ε-PLH. In contrast, the viable cell population of PL both in mono- and dual-culture biofilms treated with 64 μg/ml ε-PLH was significantly decreased (*p* < 0.05) and did not differ between two biofilm status (*p* > 0.05). More specifically, the ε-PLH treatment at 64 μg/ml achieved 4.32 and 0.85 log decrease of dual-species biofilms of LM and PL compared with 5.87 and 0.96 log reduction of individual mono-culture, respectively.

### ε-Polylysine Hydrochloride Decreased Biofilm Biomass and Exopolysaccharide Secretion

The effects of ε-PLH at the different concentrations on the biofilm biomass and exopolysaccharide production of LM and PL as mono- or dual-species biofilms are illustrated in Figures 2B,C. When treated with ε-PLH ranging from 2 to 128 μg/ml, biofilm biomass of LM and PL as mono-culture was significantly reduced at 2 and 16 μg/ml of ε-PLH (*p* < 0.05), respectively, whereas the dual-species biofilms were apparently dropped at 32 μg/ml of ε-PLH. When treated with ε-PLH at 32 μg/ml, biofilm biomass for LM and PL as mono-species reduced by 83.6 and 36.9%, respectively, which were higher than that for the dual-species biofilms (31.0%). LM failed to form the biofilm in supplement with 64 μg/ml ε-PLH. Likewise, the exopolysaccharide production of LM and PL as mono-culture was decreased in the presence of ε-PLH at 2 and 16 μg/ml, respectively, compared with LM and PL as dual-species biofilms at 32 μg/ml ε-PLH (*p* < 0.05). When treated with ε-PLH at 32 μg/ml, exopolysaccharide of LM and PL as mono- and dual-species biofilms reduced by 79.22, 27.66, and 25.84%, respectively. These findings indicated that ε-PLH effectively inhibited the biofilm development of LM as mono species in a concentration-dependent manner; however, multispecies biofilms of the two strains exhibited their community-wide resistance, resulting in higher tolerance to ε-PLH than their counterparts in mono-species biofilm.

### ε-Polylysine Hydrochloride Combined With Cinnamon Essential Oil Against Dual-Species Biofilms

**FIGURE 3:**
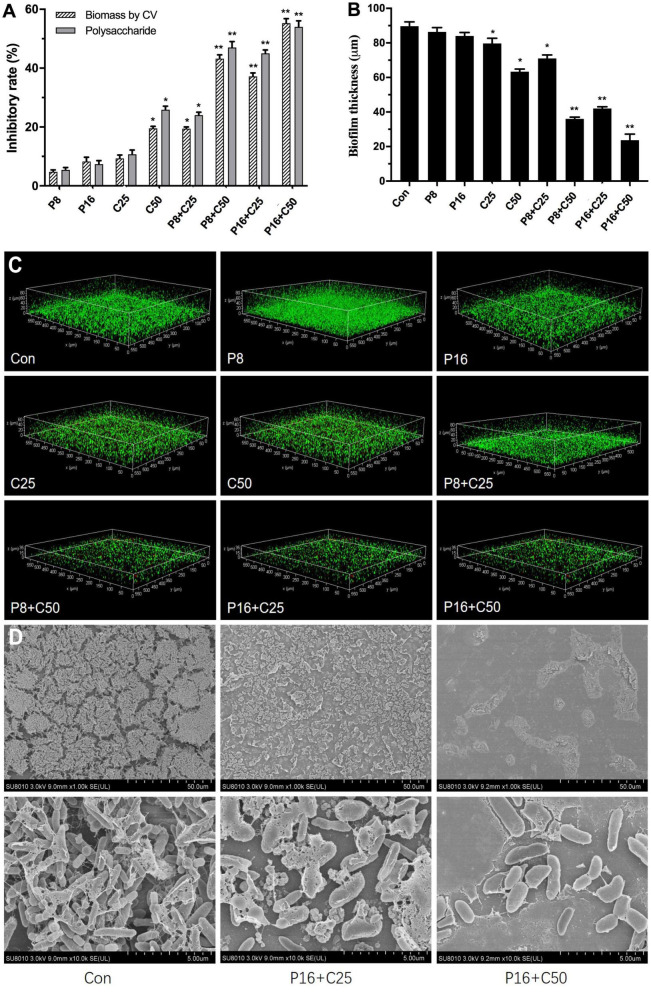
Effects of ε-PLH combined with CEOs on the biofilm formation, polysaccharides, and spatial structure of *L. monocytogenes* and *P. lundensis* as co-culture. **(A)** Biofilm biomass by CV, **(B)** polysaccharides production, **(C)** CLSM images, and **(D)** SEM images. Data were expressed as mean ± standard deviations (*n* = 3). *p < 0.05; **p < 0.01: significant interspecies difference.

Due to the high tolerance of multispecies biofilms to ε-PLH, the impacts of ε-PLH combined with CEO at the MSCs against biofilm formation as dual-species LM and PL were further assayed (Figure 3A). Compared with no effect of alone ε-PLH and CEO at two MSCs, the inhibitory effect of four combinations of ε-PLH and CEO significantly enhanced against the dual biofilms (*p* < 0.05). After incubation for 24 h, the combined treatments of P8 + C25, P8 + C50, P16 + C25, and P16 + C50 exhibited 19.4, 43.2, 37.17, and 55.27% (*p* < 0.05) decrease in CV biomass, respectively. The inhibitory rate of the three combined groups except P8 + C25 was apparently higher compared with either of the compounds used alone (*p* < 0.05). Similarly, the four combined treatments showed 24.13, 46.87, 45.03, and 54.03% reduction in exopolysaccharide levels of dual-species biofilms, respectively, in contrast to treatment of P16 (7.4%) or C50 (25.87%) used alone (Figure 3B). It was revealed that ε-PLH in combination with CEO constituents at the MSCs induced a good synergistic effect to inhibit multispecies biofilm formation of LM and PL.

### ε-Polylysine Hydrochloride Combined With Cinnamon Essential Oil Affects Dual-Biofilms Structure

We further observed the adhesion of dual-species biofilms formed by LM and PL treated with ε-PLH and CEO compounds by CLSM and SEM to assess the changes in biofilm spatial structures (Figure 3C). The two strains formed the dual biofilms with dense matrices, while the biofilm homogeneity gradually increased with the treatment of ε-PLH and CEO, as well as the decrease in viable cells. The spatial structure of dual biofilms was impaired by CEO at two MSCs in a concentration-dependent manner, in contrast to weak effect of ε-PLH at 8 and 16 μg/ml. Compared to the two compounds used alone, the four combined treatments apparently decreased the biovolume of viable cells with green signals and increased the dead cells with red signals in the dual-species biofilms. When treated with the combined groups of P8 + C25, P8 + C50, P16 + C25, and P16 + C50, the multispecies biofilm thickness was decreased to 71, 36, 42, and 23.7 μm, respectively, in contrast to 89.7 μm of thickness for dual-species biofilms control. Observations by SEM showed that many small amorphous aggregates (LM) appeared around the bacilli (PL) in the spare biofilms with less EPS matrix treated by P16 + C25 compared to control of dual-species biofilms (Figure 3D). As the concentration of combined CEO increased to 50 μg/ml (P16 + C50), few monolayers of PL adhered to surface and formed the biofilms without EPS matrix. The observations by CLSM and SEM were in line with the strong inhibition of biofilm biomass and exopolysaccharides secretion when combining ε-PLH with CEO. Thus, the combined treatments of ε-PLH and CEO at the MSCs repressed effectively adhesion and destroyed the multispecies biofilm spatial structures, indicating their synergistic inhibitory activity.

### ε-Polylysine Hydrochloride and Cinnamon Essential Oil Decrease Autoinducer-2 Activity of Co-culture

**FIGURE 4:**
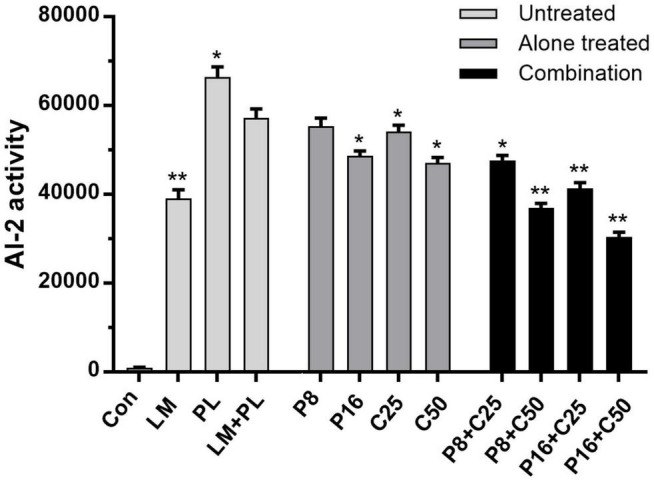
Effects of ε-PLH combined with CEO on the AI-2 activity of *L. monocytogenes* and *P. lundensis* as co-culture. Data were expressed as mean ± standard deviations (*n* = 3). *p < 0.05; **p < 0.01: significant interspecies difference.

Autoinducer-2 activities of LM and PL were performed in co-culture treated by ε-PLH or CEO, as presented in Figure 4. The supernatants of the LM and PL as mono- or co-culture induced bioluminescence in *V. harveyi* BB170, especially PL. The AI-2 activity in the co-culture of the two strains was significantly lower than that of single PL, which might be consistent with the biofilm-forming ability. Moreover, AI-2 activity of co-culture was reduced by 3.42–14.92% and 5.54–17.81% after exposure to ε-PLH or CEO used alone, while the inhibitory rates of P8 + C50, P16 + C25, and P16 + C50 were as high as 35.55–46.89%, respectively, indicating the inhibitory efficiency of combined compounds in a concentration-dependent manner (*p* < 0.05).

### ε-Polylysine Hydrochloride and Cinnamon Essential Oil Eradicate the Performed Dual-Species Biofilms

**FIGURE 5:**
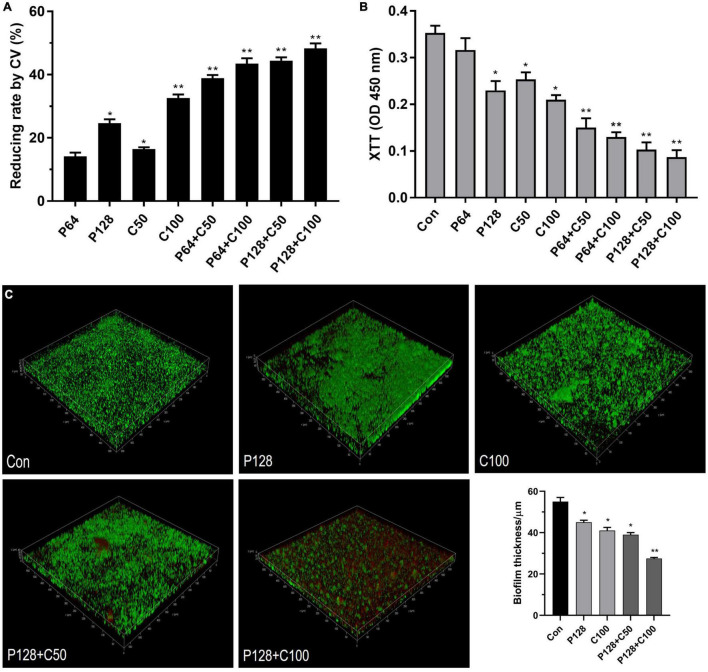
Eradication of ε-PLH combined with CEO on the performed dual-biofilms of *L. monocytogenes* and *P. lundensis*. **(A)** Biofilm removal measured by CV biomass, **(B)** biofilm inactivation measured by metabolic activity, and **(C)** CLSM images. Data were expressed as mean ± standard deviations (*n* = 3). *p < 0.05; **p < 0.01: significant interspecies difference.

The combination of ε-PLH with CEO at higher than MICs was further applied to eradicate the preformed biofilms of LM and PL as dual species after incubation for 24 h. In Figures 5A,B, biofilm removal and inactivation of combined treatments significantly increased compared to the two compounds used alone. Approximately 47.08–60.36% of dual biofilms were removed after treatment with four combined groups of P64 + C50, P64 + C100, P128 + C50, and P128 + C100 compared with 43.82% with P128 and 36.38% with C100, respectively. As expected, the combined treatments of ε-PLH and CEO resulted in the great inactivation of cellular viability in performed biofilms as dual species. The four combined treatments showed 72.46–80.5% decrease in the cell viability of 24 h performed biofilms as dual species, which were higher than P128 (36.99%) and C100 (72.05%) used alone (*p* < 0.05). Additionally, ε-PLH was effective in the biofilm removal of dual-species biofilms, while CEO exhibited the strong inactivation of biofilm cell, which could be associated with their synergistic eradication of performed biofilms. Furthermore, CLSM images also confirmed that ε-PLH in combination with CEO significantly enhanced eradication of the multispecies biofilms, in contrast to either of the compounds used alone (Figure 5C). Following the combined treatment, viable cells with green disappeared and dead cells in red increased throughout the biofilms, indicating that the dual biofilms were susceptible to combined treatments, and the combined treatment of P128 + C100 showed the highest biofilm-eradicating efficacy. In addition, the spatial structure of LM and PL as dual-species biofilms was effectively destroyed by the combined compounds, as well as the significant decrease in biofilm thickness (*p* < 0.05). More specifically, the biofilm thickness treated with P128 + C100 was decreased by nearly 50% compared to the dual-biofilms control.

## Discussion

*Listeria monocytogenes* has been isolated from various raw products and surfaces found in food processing environments following regular sanitation treatments, together with other microorganisms, mainly of spoilage and commensal microflora (Heir et al., 2019). This study evaluated the interactions of LM and PL as multispecies biofilms, as well as their sensitivity to ε-PLH in combination with CEO. It was shown that PL as mono- and dual species exhibited significantly higher biofilm formation than LM. Similarly, various *Pseudomonas* spp. readily form biofilms under the chilled conditions, such as *P. fragi* in chilled meat (Wickramasinghe et al., 2019), *P. lundensis* in pork meat (Liu et al., 2015), and *P. fluorescens* in fish (Zhu et al., 2019). Moreover, PL were here found to clearly dominate in the co-culture biofilm communities during the incubation period, rather than planktonic state. This was in agreement with the results of Papaioannou et al. (2018), who reported that *Pseudomonas* was the dominant species in mixed-species biofilm of LM. In addition, the incubation temperature showed a great increase on biofilm-forming ability of the two investigated psychrotrophs, similarly observed by Zhu et al. (2019) and Quintieri et al. (2020). For psychrotrophic species, the expression and properties of several bacterial structures, such as flagella, fimbrae, pilli, and curli, may be affected by cold temperature environment, subsequently stimulating bacterial attachment and biofilm formation (Quintieri et al., 2020).

Furthermore, the co-culture of LM with PL resulted in a significant decrease in biofilm cell and biomass, and formed the thin spare biofilm structure, which indicated that LM and PL displayed the competitive effects in the formation of dual-species biofilms. This is consistent with the findings of Puga et al. (2016, 2018), who observed a significant inhibitory effect of LM attachment when co-culture with *Pseudomonas* spp., *Escherichia coli*, and indigenous microorganisms compared with biofilms formed in individual pure cultures. The advantage of dominant PL over LM in dual-species biofilms could mainly be attributed to available space and nutrients to colonize between the two strains (Jahid and Ha, 2014; Wang et al., 2020). Recent works showed that *E. coli* O157:H7 was mainly distributed in the low layer of the dual-species biofilm, in contrast to a spoiler *P. fragi* in the top layer (Cheng et al., 2022). As obligatory aerobic and motile bacteria, *Pseudomonas* had high attachment rates and could quickly attach irreversibly to a surface and embed themselves in EPS (Mann and Wozniak, 2012). In whole multiple microbial communities, bacteria survived in top layers of multispecies were generally considered to present competitive advantages toward their counterparts occupying low layer due to easily obtained essential resources, including oxygen and nutrients (Jahid and Ha, 2014). Additionally, siderophore secreting from *Pseudomonas* could acquire iron from the environment, resulting in antagonism in the growth of neighboring microorganisms (Saha et al., 2013).

In this study, ε-PLH exhibited stronger antimicrobial and antibiofilm activity against LM than PL. Similar results were reported in *Staphylococcus aureus* (Tan et al., 2019). Like ε-polylysine, ε-PLH, as a water-soluble substance, induces structural changes in the peptidoglycan of cell wall in Gram-positive strains, causing a fragile structure of cell wall (Hyldgaard et al., 2014). Conversely, ε-PLH had a weak antibiofilm activity against PL, which could be related to strong biofilm-forming ability and high MIC of ε-PLH (Supplementary Table 1). Moreover, LM cells in the dual-species biofilm exhibited higher tolerance to ε-PLH than that in mono-species biofilm, indicating that the presence of *Pseudomonas* enhanced the survival of LM to ε-PLH. Single LM failed to form thick biofilms in the presence of shear forces, while in co-culture, the strong biofilm former *Pseudomonas* provided a protected biofilm with large EPS matrix in which LM could thrive (Fagerlund et al., 2021). Likewise, the presence of *P. aeruginosa* was showed to protect significantly *Salmonella* cells in biofilms from disinfection treatment (Pang et al., 2017). In contrast, *Pseudomonas* viable cells both in mono- or dual biofilms decreased significantly treated by 64 μg/ml ε-PLH, showing the similar resistance of two PL biofilm cells to ε-PLH. The competitive interactions of LM and PL decreased the cell adhesion and cell population of multispecies biofilms, but did not significantly influence the production of biofilm exopolysaccharide between co-culture and single PL, suggesting that the predominant species in coexistence with LM could mainly determine the structure and characteristics of the dual-species biofilms. As a shelter for bacterial community in biofilms, EPS in the multispecies biofilms could act as a diffusion-limiting barrier against the preservatives, resulting in limited compounds access to LM in the deeper layer of the biofilms (Karygianni et al., 2020). Previous studies demonstrated a competitive interaction and increased sanitizer resistance of LM when co-cultured with *Pseudomonas putida* (Saa Ibusquiza et al., 2012). The dominating competitive interactions among multispecies brewery biofilm, community members confer tolerance against the antimicrobial sulphathiazole, due to a reduction in competition upon antimicrobial treatment (Parijs and Steenackers, 2018). Conversely, *Vibrio parahaemolyticus* and LM were more susceptible to antibiotics when grown together in a dual-species biofilm due to the competitive interaction (Chen et al., 2019). These differences may be associated with several factors, such as specific strains, complex interactions, and different stresses (Chen et al., 2015). The specific mechanism of this cross-protection by the dominant species PL to LM in the multispecies biofilm community would be further explored.

Considering the high tolerance of dual-biofilms of LM and PL to ε-PLH used alone, the combination of ε-PLH and CEO was applied to inhibit biofilm formation and eradicate the performed biofilms of dual species. Interestingly, the adherent cell population and EPS produced by LM and PL as dual-species biofilms were remarkably repressed when treated with combined treatment at the MSCs, demonstrating a synergistic effect. When combined treatment of P16 + C25, many small amorphous aggregates appeared around the bacilli PL, speculating that LM biofilm cells transfer to growth state to survive in the adverse environment, such as viable-but-non-culturable state cells (VBNC). Truchado et al. (2021) reported that peroxyacetic acid and chlorine dioxide induced VBNC stage of LM and *E. coli* O157:H7 in wash water. Meanwhile, QS-mediated signaling is involved in promoting multispecies biofilms by entangling species, which are responsive toward AI-2 (Laganenka and Sourjik, 2018). Compared to single PL, quantity of AI-2 activity in the co-culture of LM and PL significantly decreased, which was positively correlated with the competition of biofilm formation as the dual-species cultures. Wang et al. (2020) indicated that DPD as precursor of AI-2 greatly promoted the development of dual-species biofilms of *P. fluorescens* and *S. aureus*. Furthermore, ε-PLH and CEO at the MSCs significantly repressed the reduction of AI-2 activity, as well as the biofilm formation as dual species, suggesting the antibiofilm activity of these compounds might be related to interference with AI-2. Indeed, cinnamon and main compound cinnamaldehyde also inhibit autoinducers of the QS system and biofilm formation without influencing bacterial growth (Vasconcelos et al., 2018).

The combined treatments of ε-PLH and CEO showed the apparent removal and inactivation of the performed biofilms of dual species, especially P128 combined with C100. The strong removal of ε-PLH indicated that the compound could effectively degrade the EPS matrix in structuring multi-biofilms, accelerating the entry of CEO into the biofilm cells. The high hydrophilicity of CEO probably enhanced the diffusion of cinnamon oil into the extracellular matrix, and targeted the inner LM and PL as dual-species biofilms by damaging cell membrane, altering the lipid profile, inhibiting ATPase (Vasconcelos et al., 2018), and causing the strong biofilm inactivation of CEO. Due to diverse modes of action affecting bacterial cells and cell targets, the synergistic combinations of ε-PLH and CEO had stronger antibiofilm activities than the compound used alone, resulting in a significant reduction in the viability of biofilm cell. Otherwise, EO effective concentration needs to be reduced to minimize consumer rejection or possible toxicological effects (Abdollahzadeh et al., 2014). The combination technology offers various advantages of using lower concentration, diminishing the formation of resistance capacity of microorganisms and achieving acceptable levels of pathogen inactivation not seen when the antimicrobial agents were applied singly (Soni et al., 2014). Combinations of thyme oil and nisin produced a high decrease in the population of LM present in minced fish meat (Abdollahzadeh et al., 2014) and minced beef (Solomakos et al., 2008) under refrigerated conditions.

## Conclusion

The finding of this study provides evidence of a competitive interaction between LM and PL in the dual-species biofilms, rather than no difference in planktonic cells. The biomass, viable cells, and thickness of dual-species biofilms were much less than of each mono-species biofilm, which could be involved in the regulation of AI-2 activity. Compared to the dominant PL, LM biofilm cells as mono-culture were sensitive to ε-PLH, but in dual-species biofilms increased its tolerance due to the protective effect of *Pseudomonas* EPS matrix. Moreover, ε-PLH and CEO displayed synergistic antibiofilm effects against the dual-species biofilms of LM and PL. Overall, the combined preservatives of ε-PLH and CEO have the potential to be developed as multi-biofilm inhibitors used in the food industry.

## Data Availability Statement

The original contributions presented in this study are included in the article/Supplementary Material, further inquiries can be directed to the corresponding author.

## Author Contributions

JZ contributed to the conception, funding acquisition, supervision, resources, and writing – review and editing. JL contributed to the data curation, investigation, methodology, software, and writing – original draft. XH contributed to the data curation, investigation, methodology, and software. YS contributed to the writing –review and editing. All authors contributed to the article and approved the submitted version.

## Conflict of Interest

The authors declare that the research was conducted in the absence of any commercial or financial relationships that could be construed as a potential conflict of interest.

## Publisher’s Note

All claims expressed in this article are solely those of the authors and do not necessarily represent those of their affiliated organizations, or those of the publisher, the editors and the reviewers. Any product that may be evaluated in this article, or claim that may be made by its manufacturer, is not guaranteed or endorsed by the publisher.
