# Supplementary material for: Synergism With ε-Polylysine Hydrochloride and Cinnamon Essential Oil Against Dual-Species Biofilms of Listeria monocytogenes and Pseudomonas lundensis
Source: Front Microbiol. 2022 Jun 10;13:885502. doi: 10.3389/fmicb.2022.885502 (PMC9226771; doi:10.3389/fmicb.2022.885502)
Supplement: Supplementary file 1 [file Table_1.DOCX]

Supplementary Table 1

MIC values of ε-polylysine hydrochloride (ε-PLH) and cinnamon essential oil (CEO) against two *Listeria monocytogenes* and *Pseudomonas*

| Strains | MIC (μg mL^-1^) | |
| --- | --- | --- |
|  | ε-PLH | CEO |
| *L.monocytogenes* ATCC19112 | 16 | 200 |
| *L.monocytogenes* ATCC19115 | 16 | 200 |
| *P. lundensis* 28 | 100 | 200 |
| *P. fluorescens* 07 | 150 | 200 |
